# Molecular targets for cystic fibrosis and therapeutic potential of monoclonal antibodies

**DOI:** 10.1016/j.jsps.2022.10.002

**Published:** 2022-10-08

**Authors:** Sivakumar S. Moni, Asmaa Al Basheer

**Affiliations:** Department of Pharmaceutics, College of Pharmacy, Jazan University, Jazan 45142, Saudi Arabia

**Keywords:** Cystic fibrosis, Molecular targets, Target-specific, Monoclonal antibodies, Therapeutic implementation

## Abstract

Cystic fibrosis (CF) is a genetic disease that affects the exocrine glands and is caused by cystic fibrosis transmembrane conductance regulator gene (CFTR) mutations. Lung disease is the leading cause of morbidity in patients. Target-specific treatment of CF has been achieved using monoclonal antibodies (mAbs). The purpose of this article is to discuss the possibility of treating CF with mAbs through their significant target specificity. We searched electronic databases in Web of Science, PubMed, EMBASE, Scopus, and Google Scholar from 1984 to 2021. We discussed the critical role of targeted therapy in cystic fibrosis, as it will be more effective at suppressing the molecular networks. After conducting a critical review of the available literature, we concluded that it is critical to understand the fundamental molecular mechanisms underlying CF prior to incorporating biologics into the therapy regimen. Omalizumab, Mepolizumab, Benralizumab, Dupilumab and KB001-A have been successfully screened for asthma-complicated CF, and their efficacies have been well reported. Despite the availability of effective targeted biologics, treating CF has remained a difficult task, particularly when it comes to reduction of secondary inflammatory mediators. This review emphasizes the overall views on CF, the immunological mechanism of CF, and the prospective therapeutic use of mAbs as potential targeted biologics for enhancing the overall status of human health.

## 1 Introduction

Cystic fibrosis (CF) is an autosomal, progressive genetic disorder that affects the lungs and digestive systems. It is one of the most common fatal autosomal recessive genetic diseases among Caucasians, and it has been estimated that it affects approximately 48,000 persons in Europe and 30,000 in the United States (Samuel et al., 2020). The disease (CF) is prevalent in Saudi Arabia and the Gulf region (Hanaa et al., 2020). Its prevalence in the Middle East has been predicted to be 1 in every 30,000–50,000 live births, with an incidence of 1 in 2000–5800 live births (Banjar and Angyalosi, 2015). According to extrapolations from known cases to the general population, the incidence among Saudi Arabian children and adolescents has been predicted to be 1 in 4243 (Samer et al., 2021). Cystic fibrosis transmembrane conductance regulator (CFTR) is a protein produced by the CFTR gene. This protein regulates the formation of mucus, sweat, saliva, tears, and digestive enzymes by acting as a channel across the cell membrane. A mutation in CFTR gene disrupts the movement of ions into and out of cells, resulting in the production of thick and sticky mucus that clogs the ducts of organs where the CFTR protein is expressed (Almughem et al., 2020). As a result, patients with CF get recurrent lung infections and impaired breathing. Furthermore, since the CFTR protein is expressed in many sites in the body, the impairment of its expression results in various clinical manifestations (Pranke et al., 2019). Although the CFTR protein affects many different sites of the body, the lungs are most frequently affected by CFTR mutation. Indeed, lung illness is the leading cause of CF-related morbidity and mortality in the United States of America (Alton et al., 2015). Chronic lung inflammation, recurrent infections and the problems with the cystic fibrosis transmembrane conductance regulator protein are the root cause of the thick, sticky mucus that is characteristic of CF patients. It is hypothesized that cystic fibrosis causes airway dryness and poor mucociliary clearance, both of which can result in tonic epithelial sodium channel (ENaC) activity. This activity drives amiloride-sensitive electrogenic sodium absorption. Reversing the drying out of the airway surface fluids can be accomplished by decreasing ENaC, which lowers sodium absorption.

Chloride is a component of salt, and the CFTR protein ordinarily forms a channel to transport chloride through the membranes of cells that line various surfaces in the body, including the surface of the lung. However, people with CF are unable to produce this channel. When the protein is not functioning properly or when it is not present at the cell surface, chloride becomes trapped within cells and is unable to attract the fluids that are necessary to hydrate the cell surface. Furthermore, the epithelial Na+ channel is likewise dysregulated in the absence of a functioning CFTR. Mucus dehydration brought on by CFTR deficiency and elevated ENaC activity results in mucus blockage, neutrophilic infiltration, and persistent bacterial infection. As a result, the cell surface becomes dehydrated. When mucus is deprived of the required fluids, it loses its wetness and becomes thick and sticky (Morrison et al., 2019, Moore and Tarran, 2018). This can cause long-term harm to the airways (Pranke et al., 2019). Defective ion transport mediated by CFTR decreases the hydration of airway surface, thereby impairing mucociliary clearance, one of primary innate immune defence mechanisms in the respiratory tract (Rafeeq and Murad, 2017). Recent research has focused on developing novel therapeutics that are rationally designed to produce a specific, long-lasting inhibition of ENaC activity in the airways while simultaneously minimizing off-target fluid transport effects, systemic exposure, and adverse effects. Methods include indirect channel-activating protease inhibitors, next-generation small molecule direct inhibitors, synthetic peptide analogs, and oligonucleotide-based treatments. These innovative medications are a significant step toward developing ENaC-directed therapies for cystic fibrosis, which is very intriguing (Shei et al., 2018). The introduction of inactivating mutant ENaC mRNA (mutENaC) inhibits endogenous heterotrimeric ENaC channels. Both in vitro and in vivo transfection of CF-based airway cells with lipid nanoparticles containing the mutENaC mutation was successful. There was a significant decrease in macroscopic ENaC currents, amiloride-sensitive ENaC currents in CF airway cells, and an increase in the airway surface liquid height (Mukherjee et al., 2020).

The therapeutic approach in CF is palliative in nature. It is basically aimed at enhancing the quality of life of the patient, while also reducing disease progression. Management strategies require long-term drug therapy for reducing inflammation, mucus clearance and pancreatic enzymes. The suppression of infection makes the requirement for antibiotics very essential. Furthermore, there is need for extensive physiotherapy along with continuous monitoring of lung function and nutritional support (Banjar and Angyalosi, 2015, Almughem et al., 2020, Rafeeq and Murad, 2017, Chaudary, 2018). The goal of CF treatment is highly based on the site of its development. The development of CF in the lungs generally controls the status of infections which depends on the sensitivity patterns of microbes to the usually prescribed antibiotics such as azithromycin, tobramycin, aztreonam, ciprofloxacin, levofloxacin, cephalexin, amoxicillin, and doxycycline. It is also very important to control inflammation using nonsteroidal anti-inflammatory drug (NSAID), steroids and cromolyn (Patrick et al., 2007). Mucus build-up in the lower airways is a hallmark of the pathogenesis of CF. In CF, the principal component of mucus is not mucin which is secreted by mucus-producing cells, but pus composed of viscous material such as polymerized DNA obtained from destroyed neutrophils (Henke and Ratjen, 2007). Mucus clearance from the airway is a critical method for a therapeutic implementation to ensure the patient's well-being and ability to breathe freely. Mucolytic drugs such as inhaled *N*-acetylcysteine and inhaled β-agonists with humidified oxygen, are beneficial in CF. Dornase alfa is an inhalation solution which improves lung function by reducing CF-induced pulmonary exacerbations (Henke and Ratjen, 2007). It has been reported that hypertonic saline (3–6 %) improved lung function of CF patients (Robinson et al., 1996). If CF develops in gastrointestinal tract (GIT), it may be managed with oral rehydration, osmotic laxatives, hyperosmolar contrast enemas, electrolyte intestinal lavage solution, or enema composed of diatrizoate meglumine and diatrizoate sodium, depending on the severity of the pathogenesis (Colombo et al., 2011). Regular administration of oral osmotic-laxative polyethylene glycol 3350 is used for 6–12 months to prevent the reoccurrence of CF (Henke and Ratjen, 2007). A mixture of pancreatic enzyme proteases, lipases and amylases is prescribed as pancreatic enzyme replacement therapy (Stern et al., 2000). In CF, dehydration is a significant pathogenic condition which can be avoided by providing optimal nutrition such as high-calorie-fat diets, extra fat-soluble vitamins A, D, E and K (ADEK), and minerals such as fluoride and zinc (Borowitz et al., 2009). The current and future therapeutic targets are mostly focused on correcting structural and functional defects in CFTR protein. Modulators of CFTR such as ivacaftor, lumacaftor and orkambi are currently prescribed in CF. Earlier reports revealed the potential benefits of using mAbs for diagnosis of CF, correction of specific CFTR mutations, and development of mAbs against *Pseudomonas aeruginosa (P. aeruginosa)*, the most common pathogen found in the lungs of CF patients (Coutinho et al., 2008, Lovato et al., 2007). The care of CF patients necessitates CFTR correction, as well as modification and rigorous symptomatic treatment aimed at reducing inflammation and infection while ensuring bronchial hydration and proper nutrition. Many therapeutic molecules have been developed for this purpose. However, they are still at the clinical trial stages. The present review provides information on the possible molecular targets, biomolecules, and drugs for treatment of CF.

## 2 Molecular basis of therapeutics

The CF is caused by a mutation in CFTR gene located on chromosome 7 (Rommens et al., 1989). The protein expressed by CFTR gene is a member of the ATP-binding cassette (ABC) protein family, a broad collection of closely related proteins with transmembrane transport functions. The CFTR protein has 2 integral membrane domains, 2 nucleotide-interacting domains, and a peculiar control region that facilitates phosphorylation-reliant gating (Akabas, 2000, Zielenski and Tsui, 1995). A mutation in CFTR gene results in a change in the protein that it normally expresses; this protein regulates the passage of salt into and out of body cells. The CFTR mutation database shows that there are more than 2,104 different mutations in the CFTR gene. However, not every all of them are responsible for CF. On the website for clinical and functional translation of CFTR (CFTR2), it is stated that 23 of the 432 CFTR gene variations detected in 89,052 CF patients did not result in the development of CF (Almughem et al., 2020). Chromosome 7 is composed of 27 exons with a 250 kb gene. According to an International Worldwide Consortium of Molecular Genetics Institutes, over 2,000 sequence variations have been found to date, with at least 200 linked to CF (Dechecchi et al., 2018). The incidence of CF among Saudi children has been estimated to be 1 in 4243, based on recorded instances (Hammoudeh et al., 2021, Zielenski, 2000, Mathew et al., 1991). Table 1 shows the prevalence of CFTR gene mutations and treatment implementation in Saudi Arabia. According to earlier reports, the most common CF-causing mutations are missense variants (42 %), nonsense variants (10 %), frameshift mutations (15 %), splicing mutations (13 %), in-frame deletion/insertion mutations (2 %), and promoter mutations (0.5 %) (Al Maghamsi et al., 2020, Banjar et al., 2021, Ryan et al., 2020, Veit et al., 2016, el-Harith et al., 1997, Tsui and Dorfman, 2013, Bobadilla et al., 2002). Mutation in CFTR gene can be classified as follows:

**Table 1:** Common Mutations of CF gene in Saudi Arabia and therapeutic implementation.

Site of mutation of CF gene	Class of mutation	Gene sequence	Potential therapies and Therapeutic implementation	References
1548delG	Frameshift mutation, Class I	c.1418delG	Translational readthrough therapy, NMD inhibitors, Bypass a specific PTC and restore mRNA levels	Banjar et al., 2021, Al Maghamsi et al., 2020, Pranke et al., 2019, Veit et al., 2016
F508del	Class II	c.1521_1523delCTT	CFTR Modulators, correct CFTR folding and trafficking to the apical PM	Banjar et al., 2021, Hanaa et al., 2020
p.Ile1234Val	Missense mutation, Class II	c.3700A > G	CFTR Modulators, correct CFTR folding and trafficking to the apical PM	Banjar et al., 2021, Hanaa et al., 2020
3120 + 1G->A	Splicing mutation Class I	c.2988 + 1G > A	Translational readthrough therapy, NMD inhibitors, bypass a specific PTC and restore mRNA levels	Al Maghamsi et al., 2020, Hanaa et al., 2020, Pranke et al., 2019, Veit et al., 2016
N1303K	Class II	c.3909C > G	CFTR Modulators, correct CFTR folding and trafficking to the apical PM	Banjar et al., 2021, Hanaa et al., 2020
S549R	Class II	c.1645A > C or c.1647 T > G or c.1647 T > A	CFTR Modulators, correct CFTR folding and trafficking to the apical PM	Banjar et al., 2021

Type/site of mutation	Class of mutation	Gene sequence	Potential therapies and Therapeutic implementation	References
H139L	Missense mutation, Class II	c.416A>T	CFTR Modulators, correct CFTR folding and trafficking to the apical PM	Hanaa et al., 2020, Ryan et al., 2020, Veit et al., 2016;
2043delG	Frameshift mutation, Class I	2043delG	Translational readthrough therapy, NMD inhibitors, bypass a specific PTC and restore mRNA levels	Hanaa et al., 2020, Veit et al., 2016, Banjar and Angyalosi, 2015
p.Gly473GlufsX54	Frameshift mutation, Class I	c.1418delG	Translational readthrough therapy, NMD inhibitors, bypass a specific PTC and restore mRNA levels	Hanaa et al., 2020, Veit et al., 2016, Banjar and Angyalosi, 2015
p.Phe508del	Frameshift mutation, Class I	c.1521_1523delCTT	Translational readthrough therapy, NMD inhibitors, bypass a specific PTC and restore mRNA levels	Hanaa et al., 2020, Veit et al., 2016, Banjar and Angyalosi, 2015
p.His139Leu	Frameshift mutation, Class I	c.1911delG	Translational readthrough therapy, NMD inhibitors, bypass a specific PTC and restore mRNA levels	Hanaa et al., 2020, Veit et al., 2016, Banjar and Angyalosi, 2015
p.Ser549Arg	Missense mutation, Class II	c.1645A>C or c.1647T>G or c.1647T>A	CFTR Modulators, correct CFTR folding and trafficking to the apical PM	Veit et al., 2016, Banjar and Angyalosi, 2015

* CFTR: Cystic fibrosis transmembrane conductance regulator protein; NMD: Nonsense-mediated mRNA decay; PTC: Premature termination codon; PM: Plasma membrane.

**Class 1:** These comprise premature stop codons encoded by Class I nucleotide sequence changes which disrupt the translation of the CFTR gene (Rowntree and Harris, 2003). Splice mutations also disrupt the synthesis of the CFTR protein (Deletang and Taulan-Cadars, 2022).

**Class 2:** This classification is based on the fact that the most prevalent mutant CFTR allele (F508del) has a deleted Phe at 508 position. This causes incorrect folding of CFTR protein and its accumulation in ER, from which it is eliminated. This mutation is characterized by a significant decrease or complete removal of the C-band and presence of the B-band (Welsh and Smith, 1993, Cheng et al., 1990).

**Classes 3 and 4:** Gating and chloride conductivity are altered by classes 3 and 4 mutations, but trafficking is unaffected. The CFTR protein normally serves as a gate at the cell surface. Examples of mutations that cause the gate to remain closed during protein synthesis are Gly551Asp, Ser549Arg, and Gly1349Asp (Fernando et al., 2016, Becq et al., 2011).

**Classes 5 and 6:** These mutations result in less functional CFTR due to defects in production and reduced protein stability, respectively. Splicing defects compromise the capacity of the cell to appropriately read the CFTR protein, thereby leaving out key sections of the gene from the resultant CFTR transcript. Other sections which are ordinarily excluded from the transcription process, wind up becoming components of the final mRNA molecule. The synthesis of a CFTR protein that is functional but not stable, i.e., it degrades too quickly once it reaches the cell surface, is caused by certain mutations in the gene (Pranke et al., 2019, Sheppard et al., 1993).

**Fig. 1:**
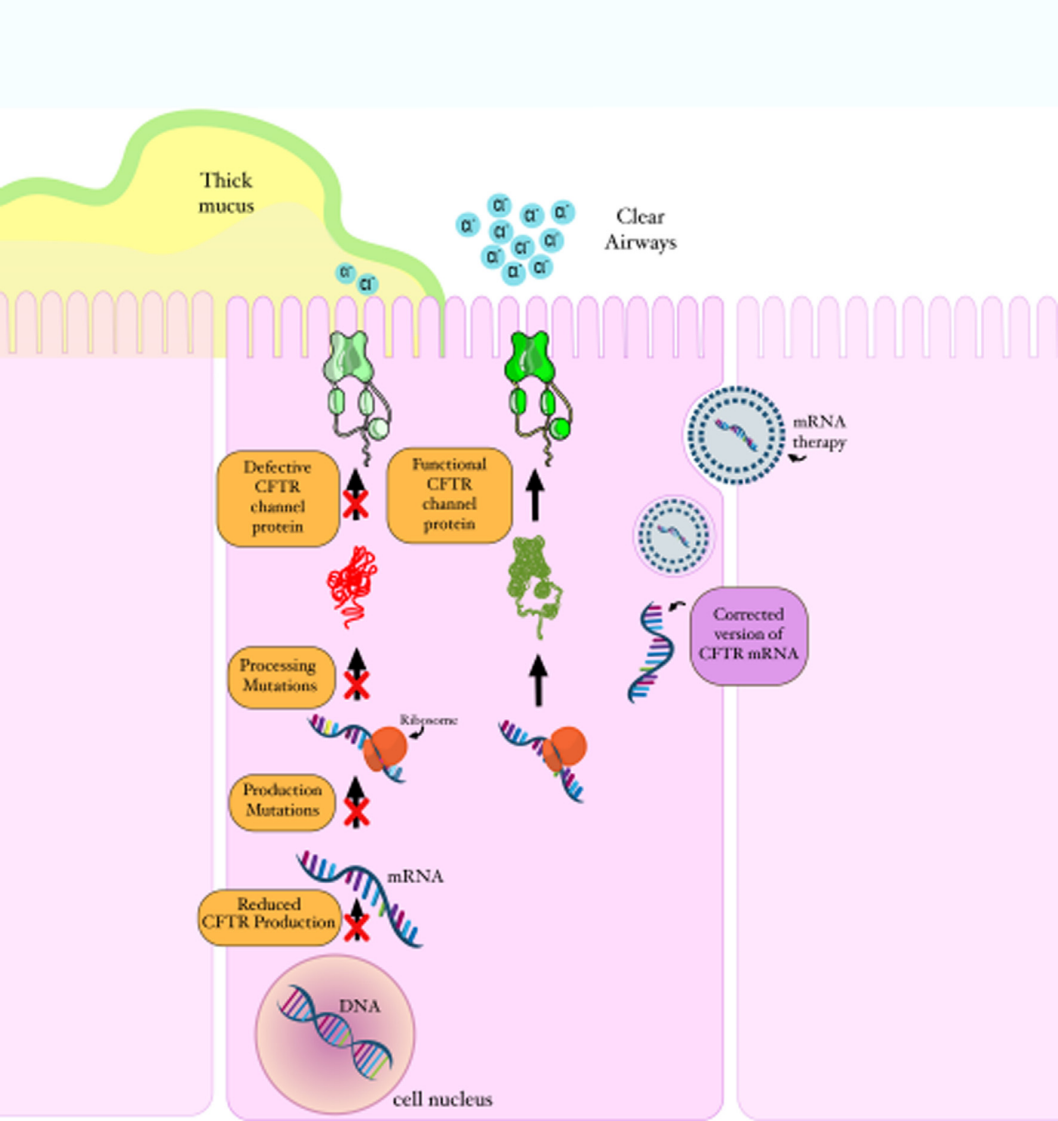
A schematic representation of CFTR mutations and mRNA therapy (Created using Adobe photoshop, version 2.5, California, USA).

The mutation in CFTR gene results in thick, sticky mucus in the respiratory, digestive, and reproductive systems, as well as an increase in the amount of sodium excreted through sweat. Over the time, the airways may become damaged (Egan, 2021, Pranke et al., 2019). The fact that CF sweat contains high sodium and chloride concentrations led to the conclusion that CF occurs as a result of improper electrolyte transport. Multiple studies found aberrant ion transport in other affected tissues, including the airways. Studies that suggested that the CFTR gene acts a chloride channel provided watershed moments in the understanding of the relationship between dysregulated ion transport and the diverse organ presentations in CF. However, the significance of CFTR in epithelial physiology has recently been shown to extend beyond its function as a Cl ^–^ channel, with numerous studies demonstrating that CFTR regulates other ion channels. As a result, CFTR is a key regulator of salt and water transport across many epithelia, and its absence causes organ-specific ion transport problems. As a result, decreased net fluid secretion across the afflicted epithelia is a common occurrence, leading to ductal blockage and organ failure. Modulators of CFTR which are involved in protein repair therapy, work on the faulty CFTR protein. These modulators include potentiators, correctors, stabilizers, and amplifiers (Egan, 2021, Mulcahy et al., 2019). The US Food and Drug Administration (US FDA) has approved the use of potentiator and corrector classes of CFTR modulators in responsive mutations (Manuella et al., 2017). Potentiators help ions move in and out of cells by facilitating the opening of CFTR channels at the cell membranes. Correctors restore the proper folding and trafficking of the CFTR protein to the cell surface (Kim and Skach, 2012). However, since they are effective only against specific mutations, they may not be viable alternatives for many patients (Manuella et al., 2017). In fact, the Saudi population has no single common variant, but rather ten different variants which account for 79 % of all CFTR variants (Hanaa et al., 2020). Another treatment option that is very appealing in CF is mRNA therapy, but it is not mutant-specific (Manuella et al., 2017). In mRNA-mediated therapy (Fig. 1), a new mRNA molecule containing the correct copy of the CFTR protein is delivered directly to the cytoplasm, allowing ribosomes to translate the mRNA molecules and produce functional CFTR protein that moves to the cell surface and functions as a chloride channel, thereby improving ion movement in and out of airway cells (Manuella et al., 2017). The possibility of treating genetic abnormalities and diseases with gene therapy is very promising. This approach offers a possibly long-lasting treatment by swapping out the genetic mutation for a “right version” of the CFTR gene. In fact, numerous research has attempted to fix the CFTR mutations using gene therapy techniques since the CF gene was discovered (Lee et al., 2021, Donnelley and Parsons, 2018). The use of inhaled siRNA therapy presents a novel therapeutic opportunity for the treatment cystic fibrosis. Nevertheless, a drug delivery mechanism that can overcome lung barriers is required to improve the efficiency of gene silencing in the airway epithelium. Recent research demonstrated that lipid-polymer hybrid nanoparticles, consisting of a core of poly(lactic-*co*-glycolic) acid and a lipid shell of dipalmitoyl phosphatidylcholine, may assist the transport of nucleic acid cargo through mucus-covered human airway epithelium. The feasibility of using non-PEGylated hybrid nanoparticles as carriers for the pulmonary delivery of siRNA in the context of the treatment of lung illness caused by cystic fibrosis. In addition, the results of this study provide a comprehensive understanding of the ways in which different models might deliver varied information regarding the interaction of nanoparticles with the mucus barrier. (Gemma et al., 2022). Gene therapy has the potential to be therapeutically beneficial in CF clinical studies that used vectors based on adenoviruses; nevertheless, improvements in vector design and delivery efficiency were necessary to address CF lung disease. Research conducted over the past nearly-three decades has shed light on the complexities of lung gene transfer and produced a huge body of data, both of which have contributed to the recent conclusion of a substantial phase study. This clinical experiment has provided the drive for additional research and development of gene transfer agents that are more powerful by demonstrating for the first time that nonviral gene transfer can stabilize lung function in CF patients. Adeno-associated viruses that delivered CFTR made it easier to understand some of the biological principles behind CFTR. Adeno-associated virus-mediated delivery of CFTR to mouse airways revealed the regulatory role of low amounts of CFTR mRNA as an activator of other chloride channels, demonstrating a connection between gene transfer and mRNA (Cooney et al., 2018, Flotte et al., 2005). The most recent development in this field is the creation of lentiviral vectors that have been specially pseudo typed to facilitate penetration into airway epithelial cells. These viral vectors show promise for the treatment of cystic fibrosis due to their ability to produce persistent expression after a single dosage as well as their capacity to be periodically delivered (Griesenbach et al., 2016). Novel improvements in gene editing technology along with cutting-edge cell models to evaluate gene engineering methods will hasten the creation of new treatments for all CF (Lee at al., 2021).

**Fig. 2:**
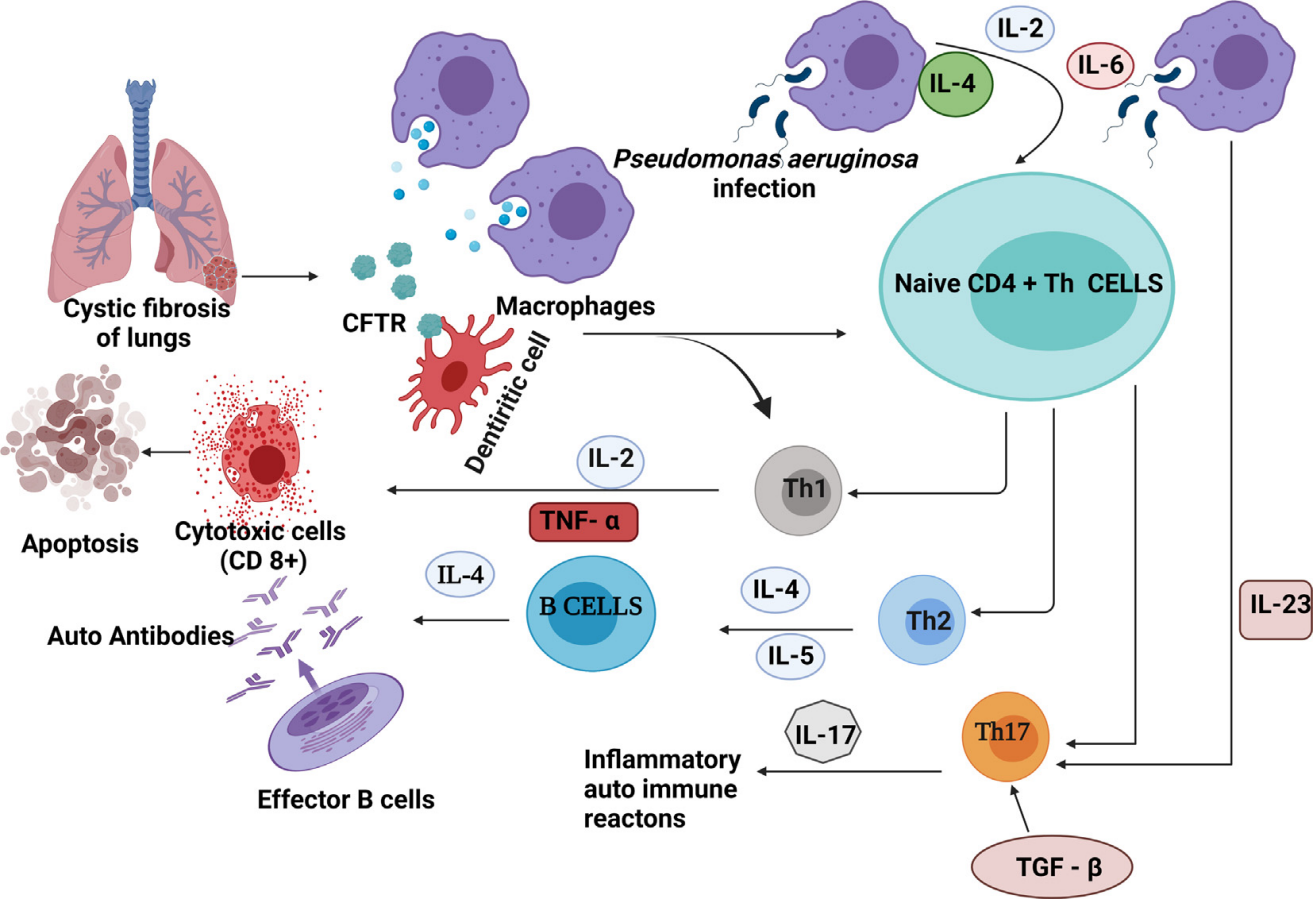
Immune mechanism in Cystic fibrosis. This figure was created with BioRender.com, Bio Render, Canada.

The use of mAbs in CF is still under investigation. Several studies in the last 30 years have investigated the potential usefulness of mAbs in detecting molecular anomalies in CF patients because of the underlying genetic abnormality. Researchers have attempted to produce mAbs that may aid the detection of structural changes in CF patients, as well as the treatment regimen. The expression of CFTR is known to occur in a variety of immune cell subtypes such as dendritic cells, monocytes/macrophages, neutrophils, and lymphocytes (Wesselkamper et al., 2008). Bacterial infections cause severe inflammatory responses in CF patients, even if they only have mild pulmonary illness (Fiona, 2009). The expressions of proinflammatory cytokines are frequently up regulated in CF patients, particularly in chronic lung infection with *P. aeruginosa* (Fig. 2). Chronic infections in CF cause lung fibrosis, and NKG2D-activated NK cells release IFN-, which aids in the clearance of *P. aeruginosa*, the most common opportunistic infection in CF (Chen et al., 2019, McGreal et al., 2010, Mitola et al., 2008). Numerous features of CF such as aberrant influx of neutrophils into the airways, cachexia, and hyperglobulinemia, may be due to the action of cytokines such as interleukin-1 (IL-1), IL-6, IL-8, and tumor necrosis factor alpha (TNF-α) (Jundi and Greene, 2015, Mitola et al., 2008, Black et al., 1998).

Supernatants of airway mucopurulent secretions were found to have mucin pro-secretory activities (SAMS) dominated by IL-1 α and IL-1 β. Like SAMS, IL-1β alone induced MUC5B and MUC5AC protein secretions and mucus hyper-concentration in CF human bronchial epithelial cells. In addition, the sterile motif-pointed domain-containing ETS transcription factor (SPDEF) and downstream endoplasmic reticulum to nucleus signaling 2 (ERN2) were activated by IL-1 β, which increased mucin gene expression, according to the findings. Increased mRNA expression levels of IL-1β, SPDEF, and ERN2 in the distal airways of excised CF lungs were related to higher MUC5B and MUC5AC expressions (Mitola et al., 2008). Despite persistent lung inflammation, it has been observed that the expression of IL-6 is low in the lung secretions of CF patients. The capacity of IL-6 to act, both at the onset and resolution of acute inflammatory responses, is thought to be due to its ability to signal through both membrane-bound and soluble receptors. In patients with CF-associated lung illness, soluble interleukin-6 receptor (sIL-6R) is a critical potentiator of IL-6 activity (Black et al., 1998). Airway inflammation, which is characterized by chronic influx of neutrophils into the airways, is a critical factor in the development of lung damage in CF patients. The most common chemokine in the lungs of CF patients is IL-8, which is inducible, thereby allowing for varying expression levels. The inducible factors include TNF-α, hyperosmotic shock, and bacterial products, for example lipopolysaccharide. Moreover, whether stimulated or unstimulated, the respiratory epithelium of CF patients produces abnormally high quantities of IL-8 (Beatriz and Andre, 2021, Zhang and Zhang, 2020; Jundi and Greene, 2015). Inflammation is regarded as the most important step in the progression of fibrosis. Interestingly, the production of pro-inflammatory cytokines, on the other hand, is not always associated with the development of fibrosis. It is known that IL-12 causes naive CD4 cells to differentiate into Th_1_ cells, which generate the pro-inflammatory cytokine IFN, which in turn reduces fibrosis by inhibiting fibroblast-induced production of collagen. As a result, Th_1_ cells are widely thought to have anti-fibrotic properties (Lammertyn et al., 2017).

The activation of inflammatory cell death pathways increases levels of TNF-α in CF patients. The TNF-α is an inflammatory cytokine produced by macrophages/monocytes during acute inflammation. It is involved in a variety of cellular signaling pathways that result in necrosis or apoptosis (Fig. 2). The TNF-α in CF airway secretions upregulates endothelial adhesion molecules and induces airway epithelial cell death (Jundi and Greene, 2015). Helper T (Th) cells play a key role in balancing healthy immune responses. Based on the prevailing pro-inflammatory/anti-inflammatory environment, CD4 + T cells may develop into Th_1_ or Th_2_ cells. These activated Th_1_ and Th_2_ cells have diverse cytokine production patterns and activities. In general, Th_1_ cells function to eradicate of internal infections, but Th_2_ cells are involved in responses against external pathogens and parasites. Uncontrolled Th_1_ responses have been linked to autoimmunity, while aberrant Th_2_ responses have been linked to the development of allergies and asthma (Cannon et al., 2003). Previously, it was reported that end-stage CF lung disease had a diverse inflammatory pattern, with increased presence of neutrophils, mast cells, CD1a and CD207 dendritic cells, as well as macrophages, CD8+ and CD4 T + cells (Cannon et al., 2003).

Chronic *P. aeruginosa* infection increases mortality in CF through aggravated generation of airway inflammation and lung damage (Wang and Wills-Karp, 2011). Interleukin-23 (IL-23), a pro-inflammatory cytokine released by macrophages during infection by *P. aeruginosa,* promotes the growth of T helper 17 (Th_17_) cells which are responsible for a variety of inflammatory autoimmune reactions. Interleukin-17 (IL-17) is a proinflammatory cytokine which regulates the production of granulocytes and the recruitment of neutrophils. Patients with CF who have exacerbated respiratory difficulties have high sputum levels of IL-17 (Qiwei et al., 2018, Wang and Wills-Karp, 2011). The CF-induced lung disease is linked to loss of pulmonary host defence which results in a vicious circle of persistent infection, inflammation, and lung tissue remodelling. The airway epithelium is critical in pulmonary host defence, and apoptosis is a physiological mechanism required for homeostasis in epithelial function. The apoptosis of defective CF epithelial cells carrying mutant versions of CFTR (van Meegen et al., 2013). The use of anti-inflammatory mAbs has also been investigated (Sivakumar and Bremnavas, 2020). However, there are still some scepticisms about their effectiveness in lowering inflammation and subsequent lung damage in CF patients, as well as their clinical value.

## 3 Monoclonal antibodies are target-specific

Non-target specific approach in the suppression of the pathological consequences of CF is a serious impediment to therapy of the disease. The mAbs generated by a single clone of B cells in response to a specific antigen. They are employed as therapeutic proteins because of their higher specificity, more effective biodistribution, very good tolerability and longer half-life than other drugs (Eraso et al., 2020). The development of new mAbs against CF will be a significant advancement in human healthcare (Farinha et al., 2004, Mendes et al., 2004). However, to generate innovative specific mAbs for the treatment of CF, it is vital to understand the pathology of the disease. The application of mAbs in the treatment of CF is still under investigation. Several studies have been conducted over many years on the possible application of mAbs in the detection of molecular aberrations in CF patients because of the underlying genetic problems (Krause et al., 2017, Collins and Varmus, 2015, Eager and Kennett, 1984).

The studies used a three-method evaluation of commercially generated mAbs to investigate their potential for detecting mutant CFTR. Methods such as Western blot, flow cytometry and confocal microscopy were used. Using these methods, it was shown that some mAbs were more effective than others in identifying mutant forms of the CFTR gene. For example, flow cytometry was successfully applied to detect the F508del and N1303K CFTR mutations using mAbs 596 and Abs 769 (Ensinck et al., 2020). On the other hand, mAbs, was able to detect mutant CFTR3 in all of these approaches (van Meegen et al., 2013). This discovery is critical in the development of tailored treatment approaches for CFTR-restoring compounds, which are currently being developed. The prospect of using mAbs to treat individuals with allergic bronchopulmonary aspergillosis who also had asthma or cystic fibrosis, has been discussed. These researchers conducted literature analysis of the trials that looked at biologics for the treatment of allergic bronchopulmonary aspergillosis in adult asthma and CF patients (Hayward et al., 1986). They reported that mAbs appeared to be more beneficial for individuals with allergic bronchopulmonary aspergillosis and asthma than for those with CF, specifically in terms of reducing the frequency of acute exacerbations and providing a steroid-sparing effect. The mAbs have been developed to facilitate the detection of structural changes in CF as well as the development of a therapy regimen. In 1982, Eager and Kennett demonstrated the binding capacity of mAbs that had been previously developed (Murao et al., 1989). They used fresh plasma from CF patients and family members to test α2-macroglobulin which was thought to target a protein that acted as a plasma protease inhibitor. They employed enzyme linked immunosorbent assay (ELISA) to determine the protein binding capacity. The results revealed a variation in binding capacity among different mAbs against α2-macroglobulin, particularly SAM94. This made them conclude that alterations in the binding ability of mAbs were mostly related to a polymorphism in α2-macroglobulin, rather than the pathophysiology of CF.

A two-site sandwich test for the detection of CF antigen was developed by generating mAbs against the CF antigen, and it was used to validate the assay (Godse et al., 2015). A study has suggested that CF protein antigen is present at high levels in the peripheral blood of CF patients (Belliveau, 2005). Despite numerous constraints such as low number of samples analysed, unpredictability of techniques of blood collection and the results obtained, and the lack of statistically significant differences between controls and CF patients in some cases, the results were positive. The specificity that may be attained using monoclonal mAbs is a powerful tool that can aid in the discovery of genetic abnormalities that are at the root of CF. On the other hand, the inflammatory pathway is responsible for the deleterious effects of CF. Therefore, the suppression of inflammatory indicators will result in a more effective therapeutic outcome, thereby minimising lung damage. The mAbs have also been explored as anti-inflammatory medicines (Table 2). However, as stated earlier, there is still a lot of doubt about the efficacy of mAbs in reducing inflammation and subsequent lung damage in CF patients, as well as their potential clinical utility.

**Table 2:** Monoclonal antibodies screened in the treatment of Cystic fibrosis.

Monoclonal antibodies	Target site	Description	Reference
Omalizumab	IgE	1. It is a recombinant humanized monoclonal anti-IgE antibody used in the treatment of Asthma and chronic urticaria. It has been approved by US FDA for human use to treat chronic urticaria. 2. The antibody down regulates FcRI expression on basophils that leads to reduced IgE level. 3. The safety and efficacy of omalizumab are not yet reported in CF patients with Asthma or Allergic bronchopulmonary aspergillosis.	Al Maghamsi et al., 2014, Eraso et al., 2020, Koutsokera et al., 2020, Belliveau, 2005, D’Amato et al., 2014
Mepolizumab	IL-5	1. Mepolizumab is a humanized monoclonal antibody of targeting IL-5. It has been approved by US FDA for the treatment of Asthma 2. It offers therapeutic benefits for eosinophilic asthma. 3. Mepolizumab is safe and easily tolerated limits type 2 inflammation.	Emma et al., 2018, Boyle et al., 2020
Benralizumab	Anti IL-5 receptor-alpha	1. Benralizumab is an immunoglobulin of the isotype IgG1k subclass monoclonal antibody. 2. It enhances antibody-dependent cell-mediated cytotoxicity. 3. It offers therapeutic effect against allergic bronchopulmonary aspergillosis in CF patients.	Yazawa et al., 2021, Canon and Modena, 2020, Seiko et al., 2019
Dupilumab	Anti IL-4 receptor-alpha	1. Dupilumab is a human immunoglobulin G4 subclass monoclonal antibody. 2. It has been approved by US-FDA for the treatment of Asthma. 3. It offers therapeutic effect against allergic bronchopulmonary aspergillosis in CF patients.	Mümmler et al., 2020

**Fig. 3:**
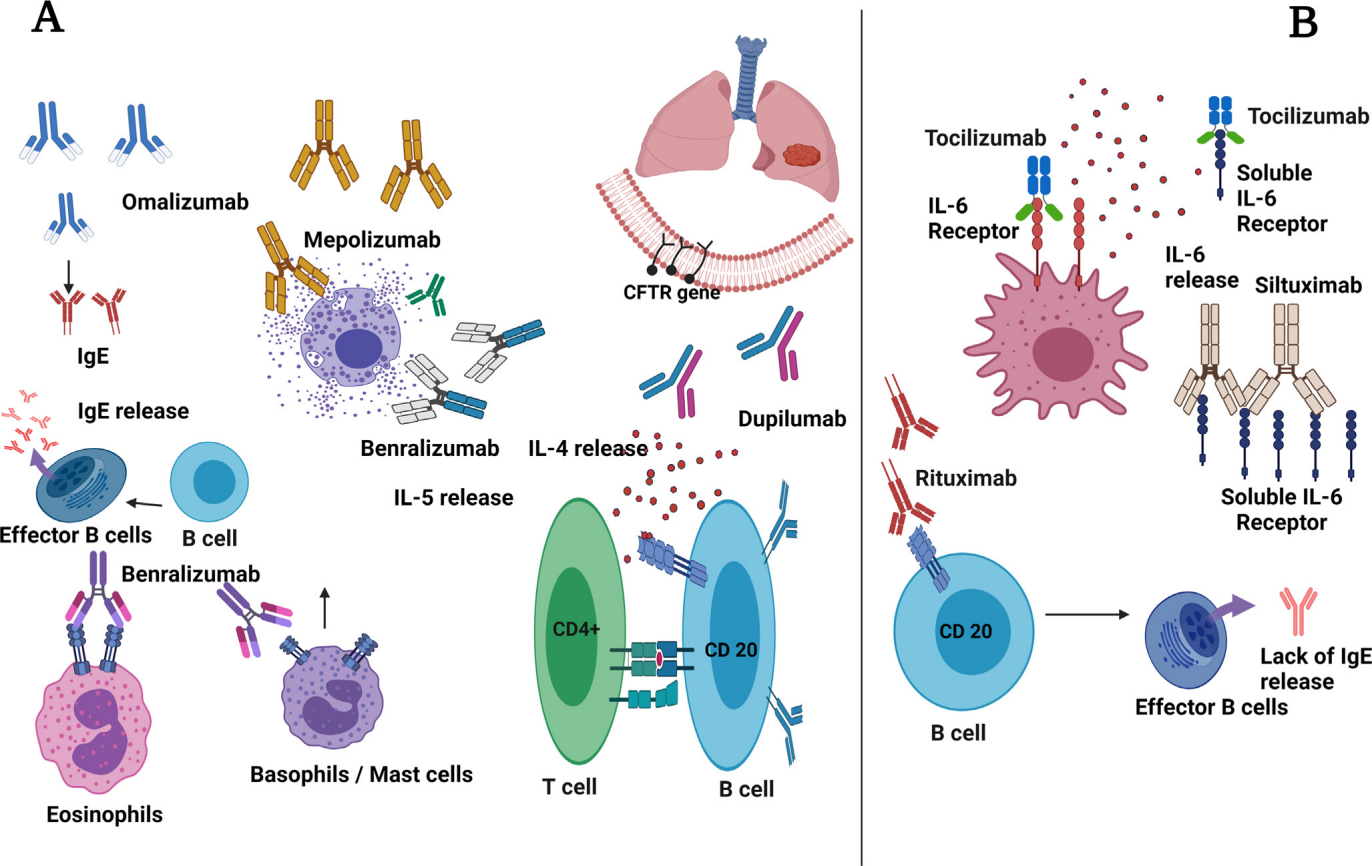
Monoclonal antibodies and their targets. (A) Monoclonal antibodies in the prime line treatment (B) Monoclonal antibodies with possible targets. This figure was created with BioRender.com, Bio Render, Canada.

The lungs of people with CF have a lot of proinflammatory cytokines and neutrophils, macrophages, and *T*-lymphocytes due to the induction of innate and adaptive immune responses. Therefore, suppressing the induction of cytokine networks will be an advantage in reducing the degree of inflammatory responses (Fig. 3).

### 3.1 Monoclonal antibody with known CF effect

#### 3.1.1 Omalizumab

Omalizumab was the first biologic drug to be licensed by the US FDA and the European Medicines Agency (EMA) for the treatment of severe atopic asthma in children, adolescents, and adults (Godse et al., 2015, Koutsokera et al., 2020, Zhang et al., 2020, Emma et al., 2018, Farne et al., 2017, D’Amato et al., 2014, Molimard et al., 2014; Belliveau et al., 2005). As a result, it is the most extensively researched biologic drug, with several studies demonstrating its efficacy and tolerability. In atopic asthma, the releases of IL-4, IL-5, IL-13 are enhanced and IgE level is increased, thereby causing eosinophilia, mucus hypersecretion and airway hyper-responsiveness (Zhang et al., 2020, Emma et al., 2018, Abonia and Philip, 2011).

Omalizumab lowers the amount of free IgE available for binding and neutralizes IgE-mediated reactions without inducing degranulation or cross-linking with IgE attached to basophils (D’Amato et al., 2014). It was recently suggested that Omalizumab should be considered for adult CF patients with asthma and allergic bronchopulmonary aspergillosis. However, not much is known about the safety and efficacy of Omalizumab (Castro-Wagner et al., 2017, D’Amato et al., 2014). An earlier investigation revealed that allergic bronchopulmonary aspergillosis occurs more frequently in CF patients who also have asthma (D’Amato et al., 2014, Emma et al., 2018). In that study, it was found that Omalizumab may be an effective alternative therapy for allergic bronchopulmonary aspergillosis in CF patients who do not react to systemic corticosteroids, or those for whom the use of the drugs is contraindicated. Furthermore, an intriguing therapeutic approach was demonstrated by the clinical trials conducted on the effectiveness of omalizumab in the treatment of allergic bronchopulmonary aspergillosis (Perisson et al., 2017). A recent clinical trial on omalizumab for the treatment of asthma or allergic bronchopulmonary aspergillosis was conducted at the Toronto adult CF center between the years 2005 and 2017. The research was conducted on 27 patients suffering from cystic fibrosis who were given omalizumab. Of these patients, 16 suffered from asthma and 11 suffered from allergic bronchopulmonary aspergillosis. According to the findings of the clinical trial, omalizumab appears to be an effective treatment for both asthma and allergic bronchopulmonary aspergillosis (Koutsokera et al., 2020).

#### 3.1.2 Mepolizumab

Mepolizumab is an anti-IL-5 monoclonal antibody used to treat patients with severe eosinophilic asthma (Ortega et al., 2014). A recent study showed that CF patients with allergic bronchopulmonary aspergillosis may also benefit from Mepolizumab (Tolebeyan et al., 2020, Boyle et al., 2020). The study found that Mepolizumab was well tolerated, and it markedly led to symptomatic improvement and clinical stability of CF patients. Another recent investigation showed that the use of Mepolizumab was more significant in specific asthma phenotype with type 2 inflammatory response (Molimard et al., 2014). Mepolizumab, injected intravenously or subcutaneously, significantly reduced asthma exacerbations in asthmatic patients (Raksha Jain et al., 2018, Milla et al., 2014). The study revealed that the degree of exacerbations was reduced by 47 % in patients who received intravenous Mepolizumab, and by 53 % in those who received subcutaneous Mepolizumab, relative to patients who received a placebo. Thus, Mepolizumab led to improvements in asthma control indicators (Canon and Modena, 2020, Seiko et al., 2019, Caruso et al., 2018). Individuals with CF who have an eosinophilic phenotype respond well to Mepolizumab which has a considerable effect on type 2 inflammation (Hector et al., 2014). Mepolizumab is a medication that is safe and well tolerated in patients with CF and type 2 inflammation (Canon and Modena, 2020). Mepolizumab seems to improve the clinical course of CF patients with a type 2 phenotype, which is marked by allergic sensitization and high levels of eosinophils. The antibody exhibited a beneficial effect on type 2 inflammatory markers, decreasing allergic inflammation markers in all CF patients (Zhang et al., 2020). Recently, the outcome of clinical trials showed that mepolizumab is a safe and focused step-up therapy for children and adolescents with severe asthma with an eosinophilic phenotype (Ullmann et al., 2022).

#### 3.1.3 Benralizumab

Benralizumab is a humanized recombinant anti IL-5 mAb which specifically binds to the alpha chain of the interleukin-5 receptor (IL-5R) expressed on eosinophils and basophils. It is beneficial for controlling eosinophilic asthma since it depletes eosinophils by increasing antibody-dependent cell-mediated cytotoxicity (Yazawa et al., 2021, Canon and Modena, 2020, Sastre and Dávila, 2018). Benralizumab has the potential to be a significant therapeutic molecule for CF patients through preservation of lung function and reduction of steroid use (Sastre and Dávila, 2018; Johana, 2017).

#### 3.1.4 Dupilumab

Dupilumab works by inhibiting the biological effects of the cytokines IL-4 and IL-13, which are important drivers of the Th2-mediated immune response. The efficacy and safety profile of Dupilumab in the treatment of allergic illnesses have been investigated for more than a decade in a variety of cohort clinical trials on asthma, atopic dermatitis, chronic rhinosinusitis with nasal polyposis, and eosinophilic esophagitis (Farne et al., 2017). Dupilumab was reported to control allergic bronchopulmonary aspergillosis in patients with asthma and CF. The study reported that the use of systemic steroids was discontinued after 3 and 6 months of treatment with Dupilumab (Mümmler et al., 2020, Ramonell et al., 2020).

#### 3.1.5 KB001-A

The drug KB001-A is a PEGylated monoclonal antibody fragment directed against *P. aeruginosa* Type III secretion. It exerts an anti-inflammatory effect by lowering the reaction of immune cells to a strain of *P. aeruginosa* that affects CF patients (Raksha Jain et al., 2018, Milla et al., 2014). Previous research on KB001-A has demonstrated that it is safe and tolerable in patients, but it no statistically significant difference has been demonstrated between CF patients who received KB001-A and those who received placebo. Earlier reports suggested that KB001-A dose-dependently decreased sputum myeloperoxidase, IL-1 and IL-8, sputum neutrophil elastase, and neutrophil count in CF patients at day 28 of study (McCracken et al., 2016).

### 3.2 Miscellaneous monoclonal antibody can be used in CF

Inflammatory response in CF patients and the use of mAbs against various biomarkers will be an added advantage in the treatment of CF patients. The potential benefits of therapeutic use of mAbs are reduction of the burden of inflammatory mediators and subsequent lung tissue damage in CF patients (Gokhale et al., 2021; Giovanni et al., 2020; Chung, 2015). The following antibodies exert unique suppressive effects on cytokine networks. However, their therapeutic efficacy against CF has not yet been reported:(Giovanni et al., 2020)

#### 3.2.1 Tocilizumab

Tocilizumab is an immunosuppressive mAb which is targeted for the treatment of rheumatoid arthritis. However, Tocilizumab reduces levels of the inflammatory marker cytokine IL-6, making it very useful in suppression of the cytokine storm Ortegwhich is a major manifestation in SARS CoV-2 viral infection (Gritti et al., 2021, Guaraldi et al., 2020, van Rhee et al., 2010). Another study found that tocilizumab, delivered intravenously or subcutaneously, lowered the risk of acute pneumonia which usually necessitates the use of invasive mechanical ventilation to prevent death of the affected patients.

#### 3.2.2 Siltuximab

Siltuximab is a recombinant chimeric monoclonal antibody which inhibits the IL-6 receptor and combats the cytokine release syndrome. Siltuximab has been reported to be an effective and safe therapeutic agent for Castleman's disease (Gritti et al., 2021, Dasgupta et al., 2013). It downregulates the expressions of IL-8 and pentraxin 3, thereby improving ventilatory efficacy in Covid-19 patients (Polverino et al., 2016).

#### 3.2.3 Rituximab

Rituximab is a chimeric monoclonal antibody which reduces inflammation and inhibits B lymphocytes by targeting the CD20 protein on their surfaces. Rituximab lowers serum levels of IgE as well as B cell activating factor, which makes it beneficial in the management of asthma. However, according to an earlier study, the use of Rituximab in the treatment of chronic obstructive pulmonary disease (COPD) has been discontinued due to increasing incidence of lung infection (Polverino et al., 2016, Dasgupta et al., 2013).

## 4 Conclusion

Treatment of CF necessitates a variety of strategies aimed at correcting the defective CFTR gene and preventing complications that may emerge as a result of the defect. There is need for further investigations on the use of mRNA and CFTR modulators as potential therapies for CF. This will aid in the development of highly effective and targeted treatments for the most frequent CF variants around the world, or to correct the faulty CTFR gene itself. Over the last decade, the therapeutic use of mAbs in clinical practice has expanded tremendously, and they have now become indispensable components of clinical practice. The mAb therapy is now crucial to treatment protocols for many diseases. Systematic deployment of mAbs to treat CF patients has major challenges, and the research is still in its early stages. Secondary inflammatory processes such as asthma and, to a lesser extent, COPD, are common complications of CF. As a result, reducing inflammation is a major focus for improving the prognosis of CF patients. Many mAbs such as omalizumab, mepolizumab, benralizumab, and dupilumab are in clinical practice for the control of severe allergic bronchopulmonary aspergillosis with asthma-complicated CF. Tocilizumab, siltuximab, and rituximab have all been shown to be effective in managing inflammation by inhibiting inflammatory markers, but their efficacy has not been tested in patients with CF and asthma. The development of mAbs for therapeutic purposes against CF has been sprouting recently, and clinical trials are showing promising outcomes. As a result, the treatment regimen should be revised to consider the potential efficacy of mAbs as targeted biologics in the treatment of CF, which will result in more effective therapeutic regimen. Since mAb treatment is highly focused on target specific, the treatment of CF also depends on the molecular targets.

## Author Contributions Statement

The two authors contributed equally to this study.

## Declaration of Competing Interest

The authors declare that they have no known competing financial interests or personal relationships that could have appeared to influence the work reported in this paper.
